# Supplementary material for: Dynamics of a Bacterial Community in the Anode and Cathode of Microbial Fuel Cells under Sulfadiazine Pressure
Source: Int J Environ Res Public Health. 2022 May 20;19(10):6253. doi: 10.3390/ijerph19106253 (PMC9141142; doi:10.3390/ijerph19106253)
Supplement: Supplementary file 1 [file ijerph-19-06253-s001.zip › ijerph-1711824-supplementary.pdf]

Supplementary Material

Dynamics of a Bacterial Community in the Anode and Cathode of Microbial Fuel Cells under Sulfadiazine Pressure

Zhenzhen Yang [1], Hongna Li [1,*], Na Li [2], Muhammad Fahad Sardar [1], Tingting Song [1], Hong Zhu [3], Xuan Xing [4] and Changxiong Zhu [1]

[1] Institute of Environment and Sustainable Development in Agriculture, Chinese Academy of Agricultural Sciences, Beijing 100081, China

[2] Department of Engineering Physics, Tsinghua University, Beijing 100084, China

[3] College of Bioscience and Resources Environment, Beijing University of Agriculture, Beijing 100096, China

[4] College of Life and Environmental Science, Minzu University of China, Beijing 100081, China

* Correspondence: lihongna828@163.com; Tel.: +86-10-8210-9561

The following are included as supplementary information for this paper:

Number of Figures: 4

Number of Tables: 2

References: 5

1. Construction of air-cathode MFCs

The MFC reactors were constructed of Plexiglas material and in a fully closed state (3.00 cm in internal diameter and 4.00 cm in length). The effective volume of each reactor was 28 mL

(Figure S1). Cathode was carbon cloth containing 0.50 mg/cm^2 Pt/C. Anode was carbon fiber brushes electrodes (the projected surface area of 7.065 cm^2) which had a two-wire Ti core served as a current collector, and brushes made of carbon fibers (Jilin Carbon Plant, China, with Young's Modulus 210-22/GPa) [1]. All brushes were first cleaned by soaking them in pure acetone overnight, then heat-treated in a muffle furnace at 450 °C for 30 min. The treatment not only facilitates the accumulation of microorganisms but also increases the actual surface area and protonated nitrogen [1]. It's an important factor for increasing power generation.

2. Determination of physicochemical parameters
3. Determination of COD by potassium dichromate titration, determination of ammonia nitrogen by Nessler reagent spectrophotometry, determination of total nitrogen by alkaline potassium persulfate ultraviolet spectrophotometry. **Bacterial community analysis**

The universal bacterial primers 338F/806R targeting the V4-V5 region of 16S rRNA were selected for the bacterial community structure analysis. The original data was spliced and filtered for quality control, and the obtained data was filtered and aggregated by Usearch software. In Usearch clustering, the analysis steps included extracting non-repeating sequences and removing single sequences without repetition. Operational taxonomic unit (OTU) clustering was classified into a minimum taxon according to the sequence that reached the similarity level of 97%. Taxonomic annotation and abundance analysis can reveal the taxonomic composition of MFCs biofilm samples. Alpha diversity and beta diversity analysis further explored the changes between different biofilm samples.

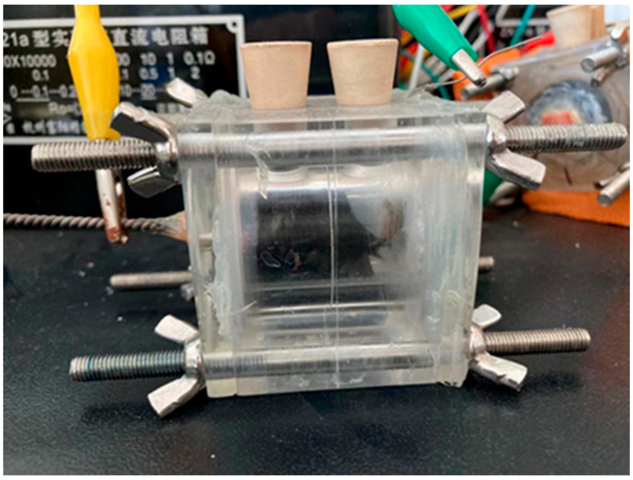

Figure S1. Construction about the air-cathode MFC reactor used in this study

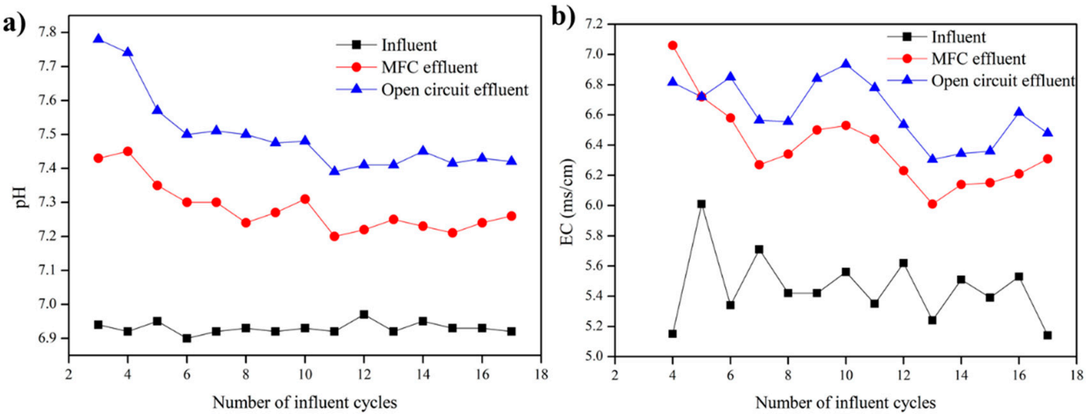

Figure S2. The physicochemical performance of air-cathode MFCs: (**a**) pH; (**b**) EC

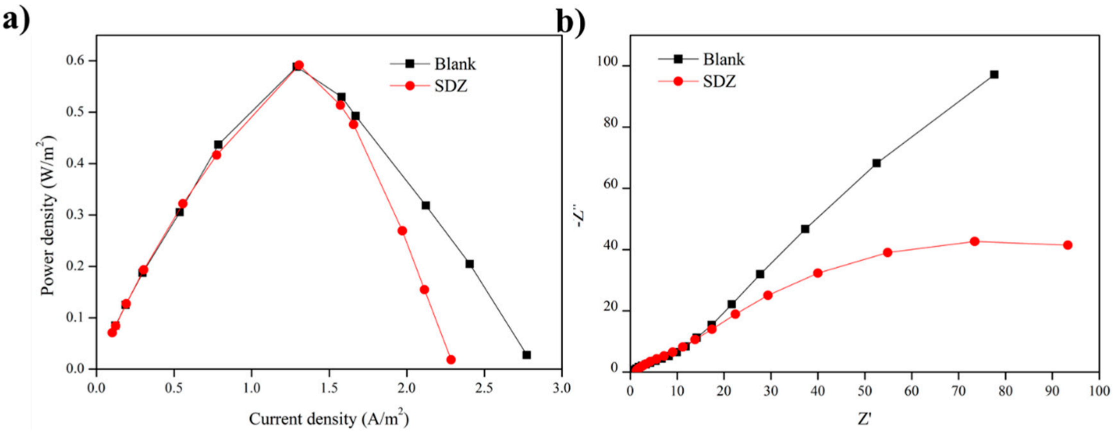

Figure S3. The electrochemical performance of air-cathode MFCs: (**a**) power density curves; (**b**) EIS polt.

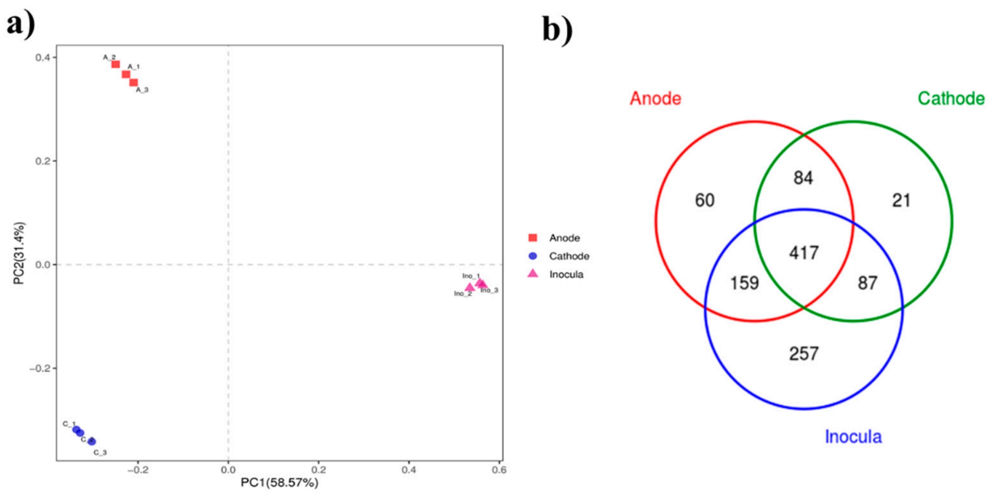

Figure S4. (**a**) PCoA on the similarity of bacterial communities in samples; (**b**) Venn diagram for the unique/shared OTUs

Table S1. The ingredients of anolyte [2,3].

Ingredient	Content/L
KCl	0.13 g
NH_4Cl	0.31 g
Na_2HPO_4	4.97 g
NaH_2PO_4	2.75 g
Sodium acetate	1.00 g
Trance minerals solution	12.5 mL
Vitamin solution	5 mL

Table S2. q-PCR primers used in this study.

Target Genes	Sequence	Amplicon Length	Annealing Temperature (℃)	Reference
16S	GCCCACTCAGTTCGATACGC<br>CGAATATGGAATCCCTAGTAACT	140	55	[4]
*int*I1	GGCTTCGTGATGCCTGCTT<br>CATTCCTGGCCGTGGTTCT	146	59	[4]
*int*I2	GTTATTTTATTGCTGGGATTAGGC<br>TTTTACGCTGCTGTATGGTGC	164	54	[4]
*sul*1	CACCGGAAACATCGCTGCA<br>AAGTTCCGCCGCAAGGCT	158	54	[5]
*sul*2	CTCCGATGGAGGCCGGTAT	190	54	[4]

References

1. Feng, Y.; Yang, Q.; Wang, X.; Logan, B.E. Treatment of carbon fiber brush anodes for improving power generation in air–cathode microbial fuel cells. *J. Power Sources.* **2010**, *195*, 1841–1844. https://doi.org/10.1016/j.jpowsour.2009.10.030.
2. Liu, H.; Logan, B.E. Electricity generation using an air-cathode single chamber microbial fuel cell in the presence and absence of a proton exchange membrane. *Environ. Sci. Technol.* **2004**, *38*, 4040–4046. https://doi.org/10.1021/es0499344.
3. Lovley, D.R.; Phillips, E. Novel mode of microbial energy metabolism: organic carbon oxidation coupled to dissimilatory reduction of iron or manganese. *Appl. Environ. Microb.* **1988**, *54*, 1472–1480. https://doi.org/10.1128/aem.54.6.1472-1480.1988.
4. Luo, Y.; Mao, D.; Ryse, M.; Zhou, Q.; Zhang, H.; Xu, L.; Alvarez, P.J.J. Trends in antibiotic resistance genes occurrence in the Haihe river, China. *Environ. Sci. Technol.* **2010**, *77*, 7220–7225. https://doi.org/10.1021/es100233w.
5. Pei, R.; Kim, S.C.; Carlson, K.H.; Pruden, A. Effect of river landscape on the sediment concentrations of antibiotics and corresponding antibiotic resistance genes (ARG). *Water. Res.* **2006**, *40*, 2427–2435. https://doi.org/10.1016/j.watres.2006.04.017.
